# Experimental infection and co-infection of dogs with *Anaplasma platys *and *Ehrlichia canis*: hematologic, serologic and molecular findings

**DOI:** 10.1186/1756-3305-3-33

**Published:** 2010-04-08

**Authors:** SD Gaunt, MJ Beall, BA Stillman, L Lorentzen, PPVP Diniz, R Chandrashekar, EB Breitschwerdt

**Affiliations:** 1 Louisiana State University, School of Veterinary Medicine, Baton Rouge, LA, USA; 2 IDEXX Laboratories, Inc. Westbrook, ME, USA; 3 Western University, College of Veterinary Medicine, Pomona, CA, USA; 4 North Carolina State University, College of Veterinary Medicine, Raleigh, NC, USA

## Abstract

**Background:**

*Rhipicephalus sanguineus *is a ubiquitous tick responsible for transmitting *Ehrlichia canis *and most likely *Anaplasma platys *to dogs, as either single or co-infections. The objective of this study was to assess the effects of either simultaneous or sequential experimental infections with *E. canis *and *A. platys *on hematological and serological parameters, duration of infection, and efficacy of doxycycline therapy in dogs infected with one or both organisms. Six dogs per group were either uninfected, *A. platys *infected, *E. canis *infected, *A. platys *and *E. canis *co-infected, *A. platys *infected and *E. canis *challenged or *E. canis *infected and *A. platys *challenged at day 112 post-infection (PI). Doxycycline treatment was initiated at 211 days PI, followed by dexamethasone immunosuppression beginning 410 days PI.

**Results:**

Initially, transient decreases in hematocrit occurred in all groups infected with *E. canis*, but the mean hematocrit was significantly lower in the *A. platys *and *E. canis *co-infected group. All dogs except the controls developed marked thrombocytopenia after initial infection followed by gradually increased platelet counts by 112 days PI in groups with the single infections, while platelet counts remained significantly lower in the *A. platys *and *E. canis *co-infected group. Both sequential and simultaneous infections of *A. platys *and *E. canis *produced an enhanced humoral immune response to *A. platys *when compared to infection with *A. platys *alone. Likewise, co-infection with *E. canis *and *A. platys *resulted in a more persistent *A. platys *infection compared to dogs infected with *A. platys *only, but nearly all *A. platys *infected dogs became *A. platy*s PCR negative prior to doxycycline treatment. *E. canis *infected dogs, whether single or co-infected, remained thrombocytopenic and *E. canis *PCR positive in blood for 420 days. When treated with doxycycline, all *E. canis *infected dogs became *E. canis *PCR negative and the thrombocytopenia resolved. Despite immunosuppression, neither *A. platys *nor *E. canis *DNA was PCR amplified from doxycycline-treated dogs.

**Conclusions:**

The results of this study demonstrate that simultaneous or sequential infection with *A. platys *and *E. canis *can alter various pathophysiological parameters in experimentally infected dogs, and because natural exposure to multiple tick-borne pathogens occurs frequently in dogs, awareness of co-infection is important in clinical practice.

## Background

*Ehrlichia canis *is a Gram-negative, obligate intracellular bacterium which infects monocytes and is the primary causative agent of canine monocytic ehrlichiosis [1]. *Rhipicephalus sanguineus *transmits *E. canis *to dogs both transtadially and intrastadially [2]. Canine infections caused by *E. canis *are more commonly reported in the southern regions of the United States, however *R. sanguineus *is distributed throughout the country [3]. Experimentally, infection with *E. canis *results in acute, subclinical and chronic disease stages with dogs having a variety of clinical signs and laboratory abnormalities including fever, lethargy, lameness, oculonasal discharge, thrombocytopenia, non-regenerative anemia, leukopenia, hyperglobulinemia and proteinuria during various stages of infection. Often, chronic infection with *E. canis *will go unrecognized because infected dogs appear healthy until late in the infection when pancytopenia, uveitis, weight loss and hemorrhagic disorders arise, and a diagnosis of ehrlichiosis is made [1].

Canine cyclic thrombocytopenia is caused by *Anaplasma platys*, a Gram-negative, obligate intracellular bacterium that infects platelets [1]. The dog is the primary reservoir host for *A. platys *and to date, this organism has not been shown to infect humans. *A. platys *is likely transmitted by the *R. sanguineus *tick, however experimental infection studies have not conclusively demonstrated transmission [4]. *A. platys *infections are often found in the same geographic regions as *E. canis *and evidence of exposure to or infection with both organisms is often detected in the same dog [5-8]. Both organisms are found on all continents throughout the world, but are more prevalent in tropical and subtropical climates [2,9]. Case reports and case series, incorporating PCR-based modalities have confirmed co-infections with *E. canis *and *A. platys *[8,10]. Experimentally, *A. platys *infections cause a cyclic thrombocytopenia that may be severe enough to result in bleeding, including petechiae and ecchymoses, but most dogs are thought to control the infection immunologically [1].

Given that *E. canis *and *A. platys *likely share the same tick vector, *R. sanguineus*, dogs may become infected with both organisms, either simultaneously or sequentially. Most dogs infested with *R. sanguineus *have numerous attached ticks. The clinical impact of an *E. canis *and *A. platys *co-infection on the pathophysiology of disease in dogs has not been thoroughly investigated. A previous study has shown that naturally infected clinically ill dogs, suspected of having either Lyme disease, granulocytic anaplasmosis, or both diseases, were nearly twice as likely to have antibodies to both *Borrelia burgdorferi *and *A. phagocytophilum *as compared to healthy dogs from the same region, suggesting that exposure to more than one pathogen may increase the likelihood of disease expression [11]. The objective of this study was to assess the effects of either simultaneous or sequential infections with *E. canis *and *A. platys *on hematological and serological parameters, the duration of infection, and the comparative efficacy of doxycycline therapy in the dogs infected with one or both organisms.

## Methods

### Inoculum

An *A. platys *isolate originating from blood of a dog with uveitis, thrombocytopenia and morulae in platelets was used to infect dogs in this study [12]. Several prior experimental infections of dogs with this isolate have been reported [13,14]. For the inoculum, blood from a splenectomized dog inoculated with this *A. platys *isolate 10 days before was collected into 3.8% citrate. Platelet-rich plasma was prepared, 10% DMSO added, and 2 mL aliquots were stored in liquid nitrogen until administration. The number of platelets containing *A. platys *inclusions or morulae in this platelet-rich plasma was approximately 35%. After storage of less than 6 months, the vials were thawed to ambient temperature and the entire 2 mL aliquot was administered within 1 hour into the cephalic vein of each dog.

The *E. canis *isolate originated from the blood of a different dog in Louisiana with fever and thrombocytopenia. Experimental canine infections, using this culture grown isolate in canine histiocytic cells, were described in two previous studies [15,16]. The *E. canis *infected cells were harvested after approximately 5 days of in vitro growth when > 80% of cells contained ehrlichial inclusions as judged by a Wright-stained cytocentrifuged smear. Ten percent DMSO was added to a suspension of the cultured *E. canis*-infected DH82 cells, after which 2 mL aliquots were stored in liquid nitrogen for less than 12 months. At the time of inoculation, the vials were thawed to ambient temperature and the 2 mL aliquot administered through the cephalic vein of each dog within 1 hour of thawing.

### Dogs

Six month old, female hound-type dogs were inoculated intravenously with *A. platys *and/or *E. canis *organisms. Six groups of six dogs each were evaluated: non-infected controls, *A. platys *infected (A), *E. canis *infected (E), *A. platys *and *E. canis *co-infected (A+E), *A. platys *infected followed by administration of *E. canis *112 days later (A→E), and *E. canis *infected followed by administration of *A. platys *112 days later (E→A). Doxycycline treatment (10 mg/kg PO daily for 28 days) was administered to half the dogs in each group beginning at 211 days post-infection (PI). To assess treatment efficacy, all dogs were subsequently immunosuppressed by administering dexamethasone 0.3 mg/kg IM daily for 5 days beginning at day 410 of the study. The timing of the challenge infection, administration of doxycycline, and dexamethasone immunosuppression was chosen based upon stabilization of platelet counts for infected dogs and the findings of a previous experimental infection using this isolate of *E. canis *[17]. The study duration was 485 days for all groups.

Whole blood and serum were collected at twice weekly, weekly or every other week intervals for 15 months after infection. Aspirates of the popliteal or prescapular lymph nodes were obtained pre- and post-immunosuppression (day 400 and 414, respectively), while bone marrow samples were obtained as aspirates collected from the iliac crest using a 16 gauge Osgood marrow needle and aseptic technique post-immunosuppression (day 414).

Physical exams that included rectal temperatures, were performed twice weekly for six weeks following each inoculation and weekly thereafter. The dogs were housed indoors in climate-controlled kennels at a facility accredited by the American Association for Laboratory Animal Science. The study was approved by the Institutional Animal Care and Use Committee (protocol #06-52) at Louisiana State University.

### Hematology

Blood was collected into 2 mL vacutainer tubes containing potassium EDTA and then quickly inverted to avoid platelet clumping. Blood samples were analyzed within 3 hours of collection using a Bayer Advia 120 to measure hematocrit, mean cell volume (MCV), mean platelet volume (MPV), and platelet and total leukocyte concentrations. Prior to analysis, each blood sample was inspected for clots; any sample with visible clots was discarded and another blood sample collected. Wright-stained blood smears were also prepared from these blood samples and reviewed for platelet aggregation. Quality control procedures for the hematology instrument included daily intralab control reagents, monthly participation in the Bayer CHECKpoint Interlab QC Program, and quarterly participation in external assurance program offered by the Veterinary Laboratory Association.

### Serology

All serum samples through day 420 were tested for antibodies to *E. canis *and *A. platys *using the Canine SNAP^® ^4Dx^® ^Test kit (IDEXX Laboratories, Inc., Westbrook, ME) according to the product insert. This multivalent ELISA (enzyme-linked immunosorbent assay) uses synthetic peptide reagents to independently detect serum antibodies to *Anaplasma *spp. (e.g. *A. platys*, *A. phagocytophilum*) and to *E. canis*. Following addition of test serum and development of the color reaction, the intensity of the color was semiquantitated by densitometry (RCP Densitometer, Tobias Associates, Ivyland, PA). The difference in optical density (OD) between the test spot color reaction and the white background on the test strip (blank) was recorded as a relative OD between 0.0 and 1.0. Although the test is not licensed for semiquantitative interpretation, a previous study has demonstrated a positive correlation between the optical density of the *E. canis *test spot and the inverse IFA titer for *E. canis *in dogs experimentally infected with *E. canis *[18].

### Polymerase Chain Reaction (PCR) Testing

Molecular evidence of infection was assessed by two independent laboratories. The first laboratory (IDEXX Laboratories, Westbrook, ME) performed real-time PCR on whole blood collected throughout the study (Days 0-154, 183, 218, 246, 275, 303, 400, 414, 420), lymph node aspirates collected pre- and post-immunosuppression, and bone marrow collected post-immunosuppression. Whole blood samples (200 μl) were processed for DNA (100 μl elution volume) using an automated system (MagNA Pure, Roche) while DNA from bone marrow and lymph node aspirates was extracted manually using a commercially available kit (HighPure Kit, Roche) according to the product insert.

A real-time PCR, hybridization probe assay was developed to detect an *A. platys p44 *polynucleotide [GenBank:GP282016] from genomic DNA [19]. Real-time PCR was performed using a LightCycler 480 genotyping master mix (Roche) in a 20 ul volume reaction with 5 ul of template DNA. Primers (Table 1) were used at a concentration of 0.3 μM for the forward primer and 0.6 μM for the reverse primer. Both probes were used at a concentration of 0.3 μM. PCR was performed under the following conditions: a single hot-start cycle at 95°C for 10 minutes followed by 50 cycles of denaturation at 95°C for 30 seconds, annealing at 58°C for 20 seconds, and extension at 72°C for 10 seconds. A melting curve was performed by heating the PCR product to 95°C for 1 minute, cooling to 45°C for one minute, and then gradually heating to 80°C. Positive samples were identified from the software as having both positive crossing points and a melting curve temperature of 66.5°C +/-1°C. Analytical sensitivity was determined to be at least 10 gene copies per reaction in negative canine genomic DNA based on serial dilutions of the control plasmid. The *A. platys p44 *PCR detected strains of *A. platys *from across the US, the Caribbean and Brazil. The *A. platys p44 *PCR did not detect *A. phagocytophilum p44 *DNA from a control plasmid containing the *A. phagocytophilum p44 *template or *A. phagocytophilum *PCR-positive field samples.

**Table 1:** Primers and probes used for the *A. platys *(Apl) and *Ehrlichia *spp. (Ehr) PCR assays [19,20].

Name	Sequence (5' to 3')
Apl forward primer	CCGGCGTTTAGTAAGATAAATG

Apl reverse primer	GCAAATTTAACGATCTCCGCC

Apl probe 1 FITC	ACAGTATCGGGGTAGCGAGAGTAGAA

Apl probe 2 LC670	GGAGATCGGCTATGAACAGTTCAAGAC

Ehr1 forward primer	CAGAGTGCTTCTCAGTGTAACGA

Ehr2 reverse primer	TCGCAGTTAAAATAGAACATGTAGTTG

Ehr3 forward primer	CAGAGTGCTTCTCAATGTAACGA

Ehr4 reverse primer	TTGCGGTTAAGATAGAACATGTAGTTG

Roche UPL probe #9	Catalog # 04685075001

An *Ehrlichia *spp. real-time PCR targeting the *groEL *gene of three *Ehrlichia *species [GenBank: U96731 (*E. canis*), AF195273 (*E. ewingii*), L10917 (*E. chaffeensis*)] was developed based on published primers [20], however using a hydrolysis probe format. Real-time PCR was performed using Lightcycler 480 probes master mix (Roche) in a 20 ul volume reaction with 200 nM of each of the four primers (Table 1) and UPL probe 9 (Roche) and 4 ul of template DNA. PCR was performed under the following conditions: a single hot-start cycle at 95°C for 5 minutes followed by 50 cycles of denaturation at 95°C for 10 seconds, annealing at 60°C for 20 seconds, and extension at 72°C for 5 seconds with a single acquisition.

Conventional PCR assays, designed to detect *A. platys*, and *E. canis *infection, were performed at the second laboratory (Intracellular Pathogens Research Laboratory at North Carolina State University) on the pre- and post-immunosuppression samples. These samples included whole blood, bone marrow and lymph node aspirates. Total DNA was automatically extracted using a Qiagen robot from 200 μl of blood with a commercially available kit (MagAttract DNA Blood kit, Qiagen, Valencia, CA). The final eluted volume was 200 μl per sample. The DNA concentration was quantified by spectrophotometry, and absence of PCR inhibitors demonstrated by the amplification of GAPDH (glyceraldehyde-3-phosphate dehydrogenase) [21]. Samples were initially screened using *16S rRNA *oligonucleotide primers designed to amplify all *Anaplasma *and *Ehrlichia *species [17]. The *E. canis *PCR assay was performed as previously described [22]. The *A. platys 16S rRNA *and *groEL *genes were targeted as described previously [11]. The limit of detection, as determined by positive control plasmid dilution for each target, was: *16S rRNA *= 10 gene copies per reaction and *groEL *gene = 5 gene copies per reaction.

In order to prevent PCR amplicon contamination, sample extraction, reaction setup, PCR amplification and amplicon detection were performed in separated areas. Negative water controls were included with each run as was a dilution of the positive control plasmid.

### Statistical analysis

The hematology data were evaluated with one-way ANOVA and Tukey's multiple comparison test to compare each group at each time point to detect significant differences (p ≤ 0.05). A software program (GraphPad Prism v.5, GraphPad Software, La Jolla, CA) was used to perform these analyses. A t-test was performed for serology and PCR results using statistical software (JMP8, SAS, Cary, NC). Agreement between the results of the two PCR assays was calculated by dividing the number of sample results in agreement by the total number of samples tested.

## Results

### Hematology and clinical signs

**Figure 1:**
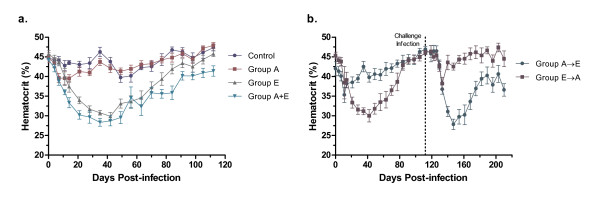
**Effect of *A. platys *and/or *E. canis *infections on hematocrits of dogs prior to doxycycline treatment**. **(a.) **Uninfected controls are compared to single infections of *A. platys *(Group A), *E. canis *(Group E) or simultaneous infections of both *A. platys *and *E. canis *(Group A+E). **(b.) **Groups receiving sequential infections of *A. platys *followed by *E. canis *(Group A→E) and *E. canis *followed by *A. platys *(Group E→A), with the challenge infection at 112 days PI (dotted line). Controls shown in panel A. (Hematocrit shown as mean ± SEM per group.)

Compared to the non-infected controls, dogs infected with *E. canis *(Group E) developed decreased hematocrits, while the hematocrits of dogs infected with *A. platys *(Group A) did not differ from controls (Fig. 1a). Dogs that were co-infected with *A. platys *and *E. canis *(Group A+E) also developed decreased hematocrits relative to the control dogs (Fig. 1a). At several time points (days 7, 84, and 112), their hematocrits were significantly lower than dogs infected with *E. canis *alone (One-way ANOVA, p ≤ 0.05). Likewise, dogs initially infected with *A. platys *and challenged with *E. canis *at day 112 (Group A→E) had a marked decrease in hematocrit following the challenge infection, whereas there was no anemia when *E. canis *infected dogs were challenged with *A. platys *at day 112 (Group E→A; Fig. 1b).

**Figure 2:**
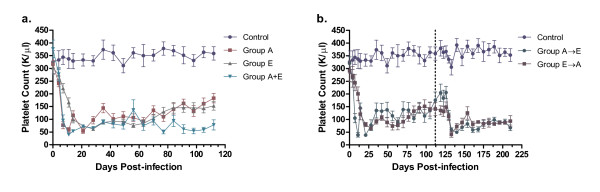
**Effect of *A. platys *and/or *E. canis *infections on platelet counts of dogs prior to doxycycline treatment**. **(a.) **Uninfected controls are compared to single infections of *A. platys *(Group A), *E. canis *(Group E) or simultaneous infections of both *A. platys *and *E. canis *(Group A+E). **(b.) **Uninfected controls are compared to groups receiving sequential infections of *A. platys *followed by *E. canis *(Group A→E) and *E. canis *followed by *A. platys *(Group E→A), with the challenge infection at 112 days PI (dotted line). (Platelet counts shown as mean ± SEM per group).

All dogs in groups A, E and A+E developed severe thrombocytopenia within 7 days compared to non-infected, control dogs (Fig. 2a). While the platelet counts in the Group A dogs (*A. platys *only) gradually increased after 75 days PI, the platelet counts in the co-infected dogs (Group A+E) remained significantly lower than Group A platelet counts at several time points (days 77, 98-130, 144-158, 171-203; One-way ANOVA, p ≤ 0.05). In comparison to *E. canis *infected dogs (Group E), the Group A+E platelet counts were also significantly lower at several time points (days 7, 11, 63, 77, 84, 120, 192; One-way ANOVA, p ≤ 0.05). In dogs that were infected sequentially with these agents (Group A→E and Group E→A), the platelet concentrations decreased following the inoculation of the second organism (Fig. 2b). However, there was no significant difference between these two groups in the severity of the thrombocytopenia, regardless of which organism was initially and subsequently administered.

Compared to the non-infected controls, the total leukocyte counts were significantly decreased in dogs from groups E and E+A between days 14-49 PI (One-way ANOVA, p ≤ 0.05), however leukocyte counts did not differ between these two groups (data not shown). The leukocyte counts of dogs infected with *A. platys *(Group A) did not differ from controls at any time point PI.

Clinically, none of the dogs developed an acute illness following inoculation with *A. platys *and/or *E. canis *organisms. Compared to controls, rectal temperatures were increased in *E. canis *infected dogs (Group E and A+E) between 21-35 days PI, but dogs infected with both *A. platys *and *E. canis *(Group A+E) were not significantly different from dogs infected with only *E. canis *(Group E).

### Real-time PCR Testing

Within three to five days PI, *A. platys *DNA was amplified by PCR in all dogs inoculated with this organism. In contrast, *E. canis *DNA was detected between seven and fourteen days PI (mean 10 days) from the *E. canis *infected dogs. All *E. canis *infected dogs consistently tested positive by PCR between day 14 and initiation of doxycycline therapy on day 211. In contrast, the majority of *A. platys *infected dogs became PCR negative prior to doxycycline treatment including those in Group E→A. Co-infection with *E. canis *(Group A+E), however, appeared to prolong the duration of active *A. platys *infection. The median duration of infection for the dogs in Group A was 104 days whereas the co-infected dogs in Group A+E had a median duration of *A. platys *infection of 119 days, excluding one dog from Group A+E that was *A. platys *PCR-positive for the duration of the study.

### Serology

On average, antibodies to *Anaplasma *spp. were first detected in the dogs inoculated with *A. platys *(Groups A and A→E) by day 16 PI (S.D. 4.4 days; range 10-24 days). On average, groups E and E→A dogs had a detectable antibody response to the *E. canis *antigens 24 days PI (S.D. 4 days, range 17-35 days). However, co-infected dogs (Group A+E) had a delayed humoral immune response to *A. platys *antigens, with *A. platys *antibodies first detectable on average 27 days PI (S.D. 10.3 days, range 14-35 days), while the *E. canis *antibody response was similar to dogs infected with *E. canis *only (avg. 24 days PI; S.D. 7.6 days). In the two groups that received challenge infections (Group A→E and Group E→A), the time between receiving the second inoculum and a measurable antibody response to antigens of the challenge infection averaged 28 days (range 14-35 days) regardless of whether the second inoculum consisted of *A. platys *or *E. canis*.

**Figure 3:**
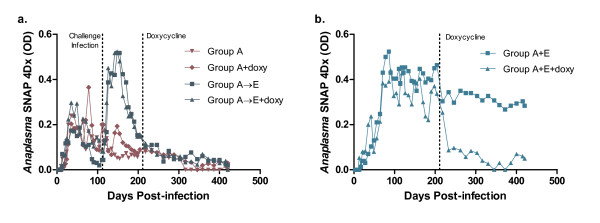
**SNAP 4Dx OD values for *Anaplasma *spp. vary with *E. canis *co-infection and doxycycline treatment**. **(a.) **Comparison of only *A. platys *infected (Group A) to *A. platys *infected challenged with *E. canis *on day 112 PI (Group A→E). Three dogs from each group were either treated with doxycycline starting at day 211 (+doxy) or not treated. **(b.) **Comparison of *Anaplasma *spp. OD values for co-infected dogs (Group A+E). Three dogs from this group were treated with doxycycline at day 211 PI (+doxy) and three dogs were left untreated. (Mean OD per group).

Serologic results from SNAP 4Dx were semiquantitated by optical densitometry for all dogs through 420 days of the study, allowing the graphical representation of the humoral immune response over time. Group A dogs, regardless of doxycycline treatment, had a steady decline in OD values after reaching an initial peak OD around 75 days PI with 5/6 dogs testing *Anaplasma *seronegative by day 420 (Fig. 3a). *A. platys *infected dogs that were subsequently challenged with *E. canis *(Group A→E) had a marked increase in their OD values for *A. platys *within two weeks of receiving the *E. canis *inoculum (Fig. 3a). Like the Group A dogs, the OD values for the Group A→E dogs, regardless of doxycycline treatment, showed a steady decline in the *Anaplasma *OD through day 420 of the study with 5/6 dogs *Anaplasma *seronegative at that time point (Fig. 3a). The serologic response to *Anaplasma *antigens was influenced by co-infection (Group A+E) such that the *A. platys *OD values were significantly greater in the co-infected group as compared to dogs in Group A between 80 and 160 days PI (t-test, p < 0.0001). Compared to the untreated co-infected dogs, *A. platys *OD values declined in the co-infected dogs receiving doxycycline therapy, with 2/3 dogs seronegative at day 420 (Fig. 3b).

**Figure 4:**
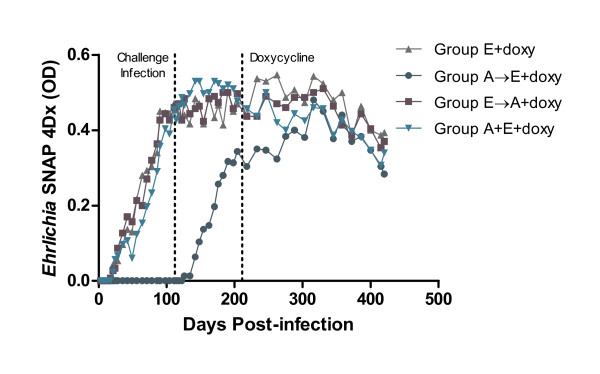
**SNAP 4Dx OD values for *E. canis *remain elevated through day 420 of the study**. Comparison of mean OD values for dogs infected only with *E. canis *(Group E), infected with *A. platys *and challenged with *E. canis *or infected with *E. canis *and challenged with *A. platys *(Groups A→E and E→A) at day 112, and dogs co-infected with *A. platys *and *E. canis *(Group A+E). Three of six dogs in each group were treated with doxycycline beginning at day 211 (+doxy) while the other three dogs per group served as untreated controls (not shown). (Mean OD per group.)

All dogs receiving the *E. canis *inoculum had a steady increase in *E. canis *OD values on SNAP 4Dx, reaching peak levels approximately 100 days PI. *E. canis *OD values remained elevated throughout the course of the study independent of doxycycline therapy (Fig. 4). Dogs in the control group remained *A. platys *and *E. canis *seronegative on SNAP 4Dx for the duration of the study.

### Doxycycline Treatment and Immunosuppression

**Figure 5:**
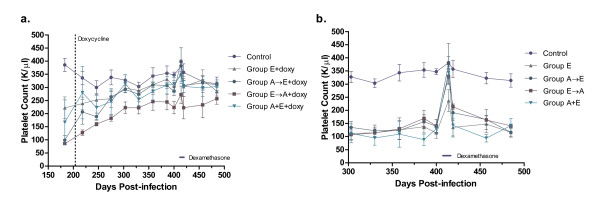
**Differences in platelet counts in dogs infected with *A. platys *and/or *E. canis *and receiving doxycycline treatment (a.) relative to their untreated controls (b)**. Three dogs from each group were either treated with doxycycline starting at day 211 (+doxy) or not treated. All doxycycline treated and untreated dogs were administered dexamethasone between days 410 and 414 PI. (Platelet counts shown as mean ± SEM per group.)

Doxycycline was administered to 3/6 dogs in each group, including uninfected controls, for a total of 28 days beginning at day 211 of the study. In order to better assess the efficacy of doxycycline following the course of treatment, all dogs were given an immunosuppressive dose of dexamethasone for 5 days beginning at day 410 of the study. Physical examinations and complete blood counts were monitored through day 485. Prior to doxycycline, the uninfected controls and Group A had platelet counts within the laboratory reference interval, while all other groups (E, A→E, E→A, and E+A) were thrombocytopenic (mean 125, 600/μl). Over the next three months, average platelet counts increased in those thrombocytopenic dogs treated with doxycycline (Fig. 5a). Dogs in groups E, A→E, E→A, and E+A that did not receive doxycycline remained thrombocytopenic at day 410 (mean 130, 500/μl; Fig. 5b). Immunosuppression with dexamethasone resulted in a transient increase in platelet counts for all dogs, but the effect was most pronounced for those dogs that were thrombocytopenic prior to immunosuppression and had not been treated with doxycycline (Fig. 5b). By day 485 of the study, the platelet counts of the dogs that did not receive doxycycline in groups E, A→E, E→A, and E+A were significantly lower than the platelet counts of the dogs in these respective groups that had been treated with doxycycline (t-test, p < 0.0001).

In addition to resolving the thrombocytopenia, doxycycline treatment appeared to successfully clear the dogs of any PCR evidence of the *E. canis *infection in blood, bone marrow and lymph node. At day 183 of the study, prior to doxycycline treatment, all dogs from groups E, A→E, E→A and E+A were PCR positive for *E. canis *DNA. Dogs treated at 99 (Group A→E) and 211 (Groups E, E→A, and A+E) days post-*E. canis *infection were *E. canis *PCR negative within 7 days of initiating doxycycline therapy and remained PCR negative for the duration of the study, including the post-immunosuppression period (Table 2). Despite immunosuppression, neither *A. platys *nor *E. canis *DNA was PCR amplified from blood, lymph node or bone marrow of any doxycycline-treated dog with complete agreement between the two laboratories performing the PCR testing.

**Table 2:** Compiled real-time and conventional *E. canis *PCR results pre- and post-immunosuppression.

	Pre-immunosuppression (Day 400)		Post-immunosuppression (Day 414 and 420)		
**Group** **(n = 3)**	**Blood**	**Lymph Node**	**Blood**	**Lymph Node**	**Bone Marrow**

Group E	3/3	3/3	3/3	2/2	2/3

Group E+doxy	0/3	0/3	0/3	0/2	0/3

Group A→E	3/3	3/3	3/3*	2/2	1/3

Group A→E+doxy	0/3	0/3	0/3	0/3	0/3

Group E→A	3/3	3/3	3/3	3/3	3/3*

Group E→A+doxy	0/3	0/3	0/3	0/3	0/3

Group A+E	3/3	3/3	3/3*	3/3	2/3*

Group A+E+doxy	0/3	0/3	0/3	0/3	0/2

Untreated control dogs are compared to the doxycycline treated dogs (+doxy). Doxycycline was administered between days 211 and 238. Dexamethasone was administered between days 410 and 414. (Number of dogs testing PCR positive/number tested in each group.*Indicates those groups where PCR results for individual samples disagreed between the methods.)

In contrast, those dogs in Groups E, A→E, E→A and E+A that were not treated with doxycycline remained *E. canis *PCR positive both pre- and post-immunosuppression in blood and lymph node, however fewer *E. canis *PCR positive results were obtained with bone marrow (Table 2). The results of both the conventional and real-time PCR assays were in complete agreement for the pre-immunosuppression samples, but demonstrated only 82% agreement on the post-immunosuppression samples. Only one untreated, *A. platys *infected dog from Group A+E tested PCR positive for *A. platys *DNA in blood pre-immunosuppression, and was PCR positive in blood, lymph node and bone marrow post-immunosuppression.

## Discussion

Results of this study demonstrate that concurrent or sequential infection with *A. platys *and *E. canis *can impact the hematological changes induced by these pathogens and can also alter the anticipated host immune response that would be induced following exposure to only one organism. Simultaneous infection with *E. canis *and *A. platys *in dogs resulted in a more pronounced anemia and thrombocytopenia, when compared to the sole infection with either pathogen. Both sequential and simultaneous infections with *A. platys *and *E. canis *produced an enhanced immune response to *A. platys *when compared to infection with *A. platys *alone. Also, co-infection with *E. canis *and *A. platys *appeared to result in a more persistent *A. platys *infection than was observed in those dogs that were infected only with *A. platys*. While the dogs in this study were infected experimentally, there is substantial evidence to support natural exposure to and infection with multiple tick-borne pathogens in dogs [5-8,10,11,23-27]. Under natural conditions, tick transmission potentially influences the course of infection and clinical manifestations, and is therefore a limitation of experimental infection studies. It is likely that co-infection or sequential infections contribute to some of the "atypical" manifestations that have been historically and clinically attributed to single pathogen infections.

In this study, the hematologic effects of infection with only *A. platys *or only *E. canis *were similar to those previously reported [28-31]. The cyclic nature of the thrombocytopenia reported in *A. platys *infected dogs was not clearly demonstrated in this study due to the comparatively low frequency (e.g. twice weekly) in which platelet concentration was measured, and due to the effect of averaging platelet concentrations from multiple dogs per study group at a point in time. When compared directly, the initial decrease in platelet concentrations (~day 10 PI) occurred more rapidly in dogs infected with only *A. platys*, as compared to dogs infected with only *E. canis*. This suggests that each of these organisms may induce pathophysiologically different mechanisms that contributed to the thrombocytopenia documented in these dogs. However, the more rapid onset of thrombocytopenia in *A. platys *infected dogs may reflect a difference in either the strain, dosage or the specific isolate of the organisms used in this study. Nevertheless, compared to *E. canis*, which induces thrombocytopenia in association with the development of anti-platelet antibodies, *A. platys *directly infects platelets and may have a more immediate effect on the platelet circulating half-life [32-34].

An unexpected alteration in the pattern of seroconversion occurred in dogs that were initially infected with *A. platys *and later challenged with *E. canis*. Following *E. canis *challenge infection, there was a dramatic increase in anti-*Anaplasma *antibodies; even for one dog in which *A. platys *serum antibodies were no longer detectable at the time of *E. canis *infection. In addition, there was no molecular evidence (PCR positivity) that *A. platys *organisms were present in the circulation of these dogs at the time this increase in *Anaplasma *serum antibodies occurred. The sensitivity and specificity of the ELISA for antibodies to *Anaplasma *and *Ehrlichia *species has also been shown to be high, reducing the likelihood of cross-reacting or false positive results [35]. This finding suggests that infection with *E. canis*, and potentially other pathogenic organisms, can induce an immunogenic effect that results in an increased anamnestic response to previously recognized antigens, in addition to a specific humoral immune response to *E. canis*. This result is potentially consistent with previous findings which demonstrated that acute *E. canis *infections do not result in immunosuppression in the dog [36].

This study is the first to report the long-term serologic and PCR results for dogs experimentally infected with *A. platys*. Previous *A. platys *experimental studies reported on the acute phase of infection; with dogs being monitored for a maximum of 75 days PI independent of treatment [29,30]. Immunological clearance of *A. platys *was supported in this study by the progression from PCR positive to PCR negative blood analyses by day 160 PI in all dogs infected only with *A. platys*. All dogs appeared to have cleared their infection prior to antibiotic treatment. These findings support prior clinical impressions that most *A. platys *strains in the United States are considered to cause minimal clinical disease, despite concurrent documentation of thrombocytopenia [37]. However, isolates from other parts of the world are reported to induce a more severe disease in dogs [5,30]. This study was limited to the strains of *A. platys *and *E. canis *available for establishing the experimental infection and the results of single, sequential and simultaneous infections may differ depending upon the strain encountered in nature. Likewise, all inoculums were prepared stored and administered in an identical manner however, undetermined variability in the infectious dose administered to each dog could have influenced the results. As dogs co-infected with *A. platys *and *E. canis *in this study developed a more persistent infection in conjunction with more severe thrombocytopenia and anemia, clinicians investigating natural infection due to *A. platys *should consider the potential influence of other known or unknown tick-borne pathogens.

In this study, similar to several previous studies utilizing experimental infections, doxycycline was found to be an efficacious therapy for *E. canis *infection when administered for four weeks [17,38]. Those dogs treated at 99 (Group A→E) and 211 (Groups E, E→A, and A+E) days post-*E. canis *infection were *E. canis *PCR negative within 7 days of beginning doxycycline therapy. For those *E. canis *infected dogs that did not receive doxycycline, *E. canis *DNA could be found through the last time point tested by PCR (day 420 of the study) with blood and lymph node samples being more reliable sources for testing than bone marrow, similar to a previous studies of naturally infected dogs [7,27].

Dexamethasone-induced immunosuppression resulted in a marked increase in the platelet counts for all dogs, which was more pronounced in chronically thrombocytopenic dogs infected with *E. canis*. Multiple mechanisms have been proposed for the thrombocytopenia associated with *E. canis *infections including increased platelet consumption, splenic sequestration and immune-mediated mechanisms associated with increased platelet destruction [1,33,39]. If immunosuppression was able to inhibit the immune-mediated destruction and the removal of platelets by tissue macrophages, the rapid rise and fall in platelet counts before and after corticosteroid administration may reflect an ongoing hyperplastic bone marrow response, which could potentially lead to hypoplastic bone marrow (exhaustion) in the chronic phase of canine ehrlichiosis [40,41]. The use of immunosuppressive corticosteroids for treatment of in immune-mediated thrombocytopenia must be considered carefully when a dog is knowingly or unknowingly infected with a pathogen. Severe immunosuppression in dogs with chronic, undiagnosed infections could contribute to highly variable clinical outcomes, including death.

## Conclusions

This study was designed to evaluate the influence of simultaneous or sequential infections with *A. platys *and *E. canis *in dogs as compared to dogs inoculated with either pathogen alone. The study identified differences in the hematological and serological parameters and in the duration of infection during simultaneous co-infection or after inducing a sequential infection. Under natural conditions, it is not always clinically possible to know the variety of organisms dogs are exposed to or infected with, particularly in those regions of the world where a spectrum of vectors and multiple pathogens are endemic. Awareness and prevention of tick-borne and other vector-borne infections, using acaracides and other preventive modalities are clearly important. Diagnostically, co-infection should be considered in those dogs with atypically severe or unusual clinical presentations.

## Competing interests

MJB, BAS, LL and RC are employees of IDEXX Laboratories, Inc. EBB is a consultant for IDEXX Laboratories, Inc. PPVD was funded as a research postdoctoral fellow in the Intracellular Pathogens Research Laboratory at North Carolina State University, which EBB directs. SG has received funding from IDEXX Laboratories, Inc. within the last 5 years.

## Authors' contributions

The authors from IDEXX have been working with Drs. Breitschwerdt, Diniz and Gaunt for a number of years on vector-borne diseases and collaborated to design, analyze and interpret the data generated in this study. MJB and SDG drafted and revised the manuscript. All authors critically reviewed and approved the final manuscript.
